# Comparison of HE4, CA125, ROMA and CPH-I for Preoperative Assessment of Adnexal Tumors

**DOI:** 10.3390/diagnostics12010226

**Published:** 2022-01-17

**Authors:** Núria Carreras-Dieguez, Ariel Glickman, Meritxell Munmany, Georgina Casanovas, Núria Agustí, Berta Díaz-Feijoo, Adela Saco, Beatriz Sánchez, Lydia Gaba, Martina Aida Angeles, Jaume Pahisa, Esther Fernández-Galán, Aureli Torné, Pere Fusté

**Affiliations:** 1 Gynecologic Oncology Unit, Clinic Institute of Gynecology, Obstetrics, and Neonatology, Hospital Clínic of Barcelona, 08036 Barcelona, Spain; ncarreras@clinic.cat (N.C.-D.); glickman@clinic.cat (A.G.); mmunmany@clinic.cat (M.M.); nagusti@clinic.cat (N.A.); bdiazfe@clinic.cat (B.D.-F.); besanchez@clinic.cat (B.S.); jpahisa@clinic.cat (J.P.); atorne@clinic.cat (A.T.); 2 Faculty of Medicine, University of Barcelona, 08036 Barcelona, Spain; 3 Medical Statistics Core Facility, Institut d’Investigacions Biomèdiques August Pi i Sunyer (IDIBAPS), 08036 Barcelona, Spain; gcasanovas@clinic.cat; 4 Gynecology Oncology Unit, Institute Clinic of Gynecology, Obstetrics and Neonatology, Hospital Clínic de Barcelona, Institut d’Investigacions Biomèdiques August Pi i Sunyer (IDIBAPS), Faculty of Medicine, University of Barcelona, 08036 Barcelona, Spain; 5 Institut de Salut Global de Barcelona (ISGlobal), 08036 Barcelona, Spain; masaco@clinic.cat; 6 Gynecology Oncology Unit, Department of Pathology, Hospital Clínic of Barcelona, 08036 Barcelona, Spain; 7 Gynecology Oncology Unit, Department of Medical Oncology, Hospital Clínic of Barcelona, 08036 Barcelona, Spain; lgaba@clinic.cat; 8 Translational Genomics and Targeted Therapeutics in Solid Tumors, Institut d’Investigacions Biomèdiques August Pi i Sunyer (IDIBAPS), 08036 Barcelona, Spain; 9 Department of Surgical Oncology, Institut Claudius Regaud, Institut Universitaire du Cancer de Toulouse (IUCT), Oncopole, 31100 Toulouse, France; angeles@clinic.cat; 10 Biomedical Diagnostic Center, Hospital Clínic of Barcelona, 08036 Barcelona, Spain; esfernandez@clinic.cat

**Keywords:** tumor markers, HE4, Ca125, ROMA, CPH-I, adnexal tumors

## Abstract

(1) OBJECTIVE: To assess the performance of CA125, HE4, ROMA index and CPH-I index to preoperatively identify epithelial ovarian cancer (EOC) or metastatic cancer in the ovary (MCO). (2) METHODS: single center retrospective study, including women with a diagnosis of adnexal mass. We obtained the AUC, sensitivity, specificity and predictive values were of HE4, CA125, ROMA and CPH-I for the diagnosis of EOC and MCO. Subgroup analysis for women harboring adnexal masses with inconclusive diagnosis of malignancy by ultrasound features and Stage I EOC was performed. (3) RESULTS: 1071 patients were included, 852 (79.6%) presented benign/borderline tumors and 219 (20.4%) presented EOC/MCO. AUC for HE4 was higher than for CA125 (0.91 vs. 0.87). No differences were seen between AUC of ROMA and CPH-I, but they were both higher than HE4 AUC. None of the tumor markers alone achieved a sensitivity of 90%; HE4 was highly specific (93.5%). ROMA showed a sensitivity and specificity of 91.1% and 84.6% respectively, while CPH-I showed a sensitivity of 91.1% with 79.2% specificity. For patients with inconclusive diagnosis of malignancy by ultrasound features and with Stage I EOC, ROMA showed the best diagnostic performance (4) CONCLUSIONS: ROMA and CPH-I perform better than tumor markers alone to identify patients harboring EOC or MCO. They can be helpful to assess the risk of malignancy of adnexal masses, especially in cases where ultrasonographic diagnosis is challenging (stage I EOC, inconclusive diagnosis of malignancy by ultrasound features).

## 1. Introduction

Epithelial ovarian cancer (EOC) is the leading cause of death in patients with gynecological malignancies [1]. Most cases are diagnosed in advanced stages, since screening has not shown to be beneficial, and the disease has an insidious onset with unspecific presenting symptoms. Pelvic adnexal masses are common in female population, yet only a small percentage represent ovarian malignancies [2]. While benign ovarian masses can be managed in non-specialized centers, patients with EOC or metastatic cancer in the ovary (MCO) should be treated by a multidisciplinary experienced team [3]. Surgical staging for suspected early-stage EOC is a complex procedure. Moreover, it has been demonstrated that, in advanced EOC, there is an increase in overall survival when cytoreductive surgery is performed by a specialized team in gynecologic oncology [3]. Therefore, an accurate differential diagnosis and referral to specialized centers of women harboring suspicious adnexal masses is critical to enhancing their survival.

Preoperatively, ultrasound and serum tumor markers can help to identify patients with adnexal masses that harbor a high risk of EOC or MCO. Expert ultrasonography examination plays an important role in the detection of EOC [4,5], but it is challenging in early stages and is not available in all centers. IOTA simple rules are 10 validated ultrasound-based rules allowing to classify adnexal masses in benign, malignant, or unclassifiable; several studies have reported a prevalence of unclassifiable adnexal masses of 22–33% [6,7,8]. The serum tumor marker cancer antigen 125 (CA125) has been traditionally used as a tool for diagnosis and follow-up for EOC patients [9]. However, CA125 serum levels are increased in less than half of early-stage EOC cases, and also raise in many other benign or malignant medical conditions [10], resulting in a decreased sensitivity and specificity [11]. Human epididymis protein 4 (HE4) is a serum tumor marker introduced during the last decade for EOC diagnosis. It has shown to improve sensitivity and specificity for detection of EOC over CA125, especially in premenopausal women and in early stages of EOC [11]. Unlike CA125, HE4 is not overexpressed in benign ovarian disease or normal ovarian tissue [12]. HE4 levels increase progressively with advancing age, which raises an issue when defining its reference range [13]. Serum HE4 levels have shown to be increased in other pathological conditions such as lung cancer, endometrial cancer, or renal failure [14].

Several models have been developed to determine the risk of malignancy of adnexal masses. Such tools are based on different combinations of clinical data, ultrasound features, and serum tumor markers. The Risk of Ovarian Malignancy Algorithm (ROMA), described in 2009 by Moore et al. [15], combines serum levels of HE4 and CA125 with menopausal status to obtain a probability risk of harboring EOC. A recent meta-analysis has shown a sensitivity of 87% and a specificity of 86% [16]. In 2015, Karlsen et al., developed the Copenhagen Index (CPH-I), based as well on serum HE4 and CA125, but combined with age [17]. CPH-I model has shown a sensitivity of 69% and specificity of 85% to differentiate benign from malignant and borderline tumors [18].

Several studies evaluating the role of HE4, CA125, and ROMA in preoperative assessment of adnexal masses have been published [14,16,19,20,21]. However, scarce literature regarding the performance of CPH-I can be found [18,22,23]. Furthermore, there is little information about the role of ROMA and CPH-I in the evaluation of specific subgroups of patients in which the differential diagnosis of adnexal tumors is particularly challenging, such as women harboring adnexal masses with an inconclusive diagnosis by ultrasonographic examination, women with Stage I EOC (whose diagnosis is difficult by ultrasound [24], or premenopausal women, in which tumor markers have shown worse performance compared to postmenopausal women [19]). Thus, it remains unclear which is the best approach for preoperative evaluation of adnexal masses.

The main objective of this study was to assess the performance of CA125, HE4, ROMA, and CPH-I on the specific detection of adnexal masses who will benefit from derivation to a reference center, prioritization in surgery waiting list and surgery performance by surgeons specialized in gynecologic oncology, that is EOC and MCO. Our secondary objectives were to evaluate the role of CA125, HE4, ROMA, and CPH-I to diagnose EOC or MCO in three challenging situations: premenopausal women, Stage I EOC and adnexal masses with an inconclusive diagnosis of malignancy by ultrasound features, using IOTA simple rules.

## 2. Materials and Methods

We performed an observational retrospective study, including women consecutively referred to the Gynecological Oncology Unit of Hospital Clínic de Barcelona with a diagnosis of ovarian cyst or pelvic mass between January 2000 and December 2018. Inclusion criteria were: (1) patients with an ovarian cyst or pelvic mass identified in pelvic imaging (ultrasound, CT scan, RMI); (2) tumor markers and ultrasound performed preoperatively; (3) pathology results available and evaluated in our center, confirming benign ovarian disease, borderline epithelial ovarian tumors, EOC or MCO. Patients without pathology results available or harboring other synchronic malignancies were excluded. Patients harboring non-epithelial ovarian cancer or non-ovarian masses were excluded from the analysis.

We defined two study groups according to pathology findings. Group A comprised women harboring benign pathology and borderline epithelial ovarian tumors. Group B included women diagnosed with EOC or MCO. This classification was based on the differences in the management of adnexal masses between these groups: patients with EOC or MCO benefit from derivation to a reference center, prioritization in surgery waiting list and surgery performance by surgeons specialized in gynecologic oncology.

Data were collected from clinical records. The preoperative results of blood tests were retrieved from medical records. Serum levels of CA125 and HE4 were measured using a chemiluminescent enzyme immunoassay on the ADVIA Centaur^®^ XP (Siemens Healthcare Diagnostics Inc., Tarrytown, NY, USA) and the Architect^®^ Analyzer (Abbott Laboratories, Chicago, IL, USA), respectively; as part of our institution’s usual clinical practice. All samples were stored, and the tests were performed according to the manufacturer’s instructions; the controls were within the ranges provided. HE4 was not considered in patients with impaired kidney function (serum creatinine levels higher than 1.3 mg/dL or glomerular filtration rate minor to 90 mL/min). Index values (ROMA and CPH-I) were computed for all patients according to the published algorithms [15,17]. Menopause was defined as cessation of menstruation for at least 12 months. Transvaginal ultrasound by an expert ultrasonographer is always performed preoperatively in patients harboring an adnexal mass as part of our institution protocol. Adnexal masses were classified as probably benign, probably malignant, or inconclusive diagnosis according to IOTA simple rules [6]. All the included patients underwent surgery after diagnosis of the adnexal mass. Patients with suspected malignancy underwent surgery less than 1 month after diagnosis, whereas patients without suspected malignancy underwent surgery less than 3 years after diagnosis. Pathology analysis of removed or biopsied adnexal masses was performed according to 2014 WHO classification of tumors of the female reproductive organs [25]. The value of tumor markers was available to pathologists at the time of evaluation of adnexal masses.

Categorical variables were reported as absolute frequencies (*n*) and proportions (%), and continuous variables as mean ± standard deviation or median [interquartile range]. The receiver operating characteristic (ROC) curves for tumor markers, CPH-I and ROMA were plotted, and the diagnostic accuracy, sensitivity, specificity, positive predictive value (PPV) and negative predictive value (NPV) and their 95% confidence intervals (CI) were calculated to evaluate the diagnostic performance of CA125, HE4, ROMA, and CPH-I at different cut-off points reported by literature [15,16,17,18,22]. AUCs were compared using the Delong nonparametric approach [26]. We performed a subgroup analysis including only premenopausal, postmenopausal women, Stage I EOC, and women harboring adnexal masses with an inconclusive diagnosis of malignancy by ultrasound features using IOTA simple rules [27].

Statistical analysis was performed using STATA/IC version 15.1 (College Station, TX, USA) and SAS version 9.4 (SAS Institute Inc., Cary, NC, USA). The level of significance was set at two-sided 5% (i.e., 0.05).

## 3. Results

During the study period, 1124 patients were referred to our center because of adnexal masses. We excluded 31 patients because serum tumor markers had not been obtained preoperatively, three patients because they presented other synchronic malignancies, and four patients because of surgical findings of non-adnexal pathology. Twelve patients had impaired kidney function. Sixteen patients had non-epithelial ovarian cancer and were excluded as well. Finally, 1071 patients were included. Table 1 summarizes the main characteristics of the studied population.

Eight-hundred fifty-two (79.6%) patients presented benign or borderline epithelial tumors (Group A) and 219 (20.4%) presented EOC or MCO (Group B). One hundred and ninety-seven patients harbored EOC, fifty-eight of whom had FIGO Stage I. Serous carcinoma was the most frequent type of EOC, followed by endometrioid carcinoma. Twenty-two patients (2.05%) harbored MCO. Seventy-four patients had epithelial borderline tumors. Premenopausal women accounted for 58.7% of the sample, while there were 41.3% postmenopausal women. In 348 patients (32.58%), there was an inconclusive diagnosis by ultrasound features using IOTA simple rules. The mean age of patients in Group A was significantly lower that in Group B (44.8 ± 0.53 vs. 59.6 ± 0.88 years, *p* < 0.05). The proportion of premenopausal patients was higher in Group A (67.3% vs. 25.1%). There were no differences in kidney function (creatinine levels) between study groups (*p* < 0.19).

For Group B patients’ median values of CA125 and HE4 were 184 (49–671) U/mL and 246 (104.7–765.5) pmol/L, respectively. For Group A patients, they were 13 (8- 30) U/mL and 45.5 (35.4–57.1) pmol/L. The difference between groups was statistically significant for both tumor markers. The predicted probability of malignancy of ROMA (0.85 vs. 0.07) and CPH-I (0.68 vs. 0.01) was also higher in Group B. No adverse events derived from tumor marker testing were registered.

**Figure 1:**
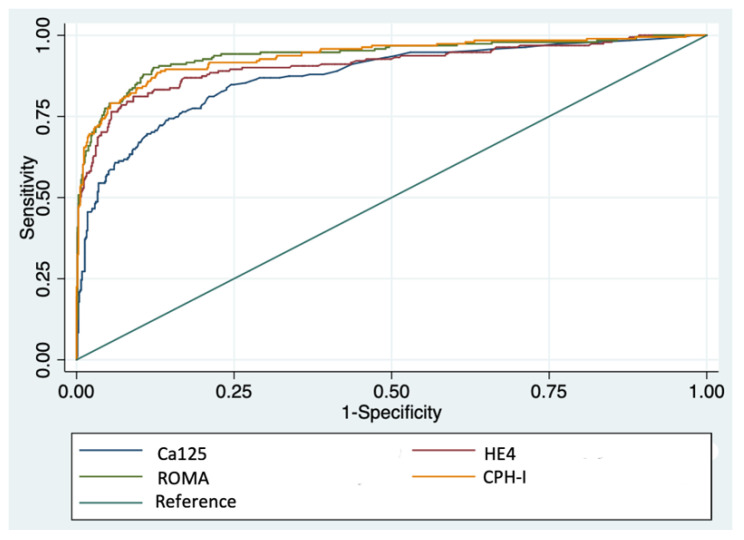
ROC curve of Ca125, He4, ROMA and CPH for diagnosis of EOC or MCO.

Table 2 and Figure 1 show the results of ROC analysis for detecting EOC or MCO. The area under the curve (AUC) for HE4 was significantly higher than AUC for CA125 (*p* < 0.05). Non-significant differences were seen between the AUC of ROMA and CPH-I, but they were both higher than HE4 AUC (*p* < 0.05). Subgroup analysis showed that in premenopausal women, HE4 performed better than CA125 (*p* < 0.05), and was equivalent to ROMA or CPH-I. In postmenopausal women, HE4 and CA125 AUCs showed no differences, but both ROMA and CPH-I performed better than the tumor markers alone (*p* < 0.05). Considering only adnexal masses with an inconclusive diagnosis using IOTA simple rules, ROMA performed significantly better than CPH-I and tumor markers alone (*p* < 0.05). With regard to the diagnosis of Stage I EOC, ROMA and CPH-I performed similarly, and ROMA performed significantly better than CA125 and HE4 alone (*p* < 0.05). We found no significant differences in the AUC of HE4 and CPH-I in Stage I EOC. Supplemental Table S1 summarizes the main findings of ROC analysis.

Table 3 shows sensitivity, specificity, and PPV and NPV of CA125, HE4, ROMA, and CPH-I at different cutoff points previously reported by literature. None of the tumor markers alone achieved a sensitivity of 90%; HE4 at a cutoff point of 70 for premenopausal women and 140 for postmenopausal women, was highly specific (93.5%). ROMA index at a cutoff point of 15 showed a sensitivity and specificity of 91.1% and 84.6% respectively and of 91.6% and 82.6% at a cutoff point of 12.5/14.4 for pre/postmenopausal women; in both cases, PPV was higher than 50%. CPH-I at a cutoff point of three showed a sensitivity of 91.1% with 79.2% specificity and PPV of 54.72%.

False negative results for CA125, HE4, ROMA and CPH-I were found in 53, 38, 8 and 20 patients, respectively. Supplemental Table S2 summarizes histological tumor types of patients with false negative results. For CA125 and HE4, most patients with false negative results harbored serous carcinoma. The most common false negative result for ROMA and CPH-I was obtained in patients with metastatic cancer in the ovary. More than 70% of patients with false negative results of tumor markers or probabilistic indexes suffered from early-stage EOC. Regarding false positive results, 62.7% (*n* = 96) of patients with false positive result for CA125 determination harbored endometriomas. This proportion was lower for ROMA (14.1%), CPH-I (19.35%) and HE4 (3.7%, *n* = 1). Nineteen (9.9%) patients in Group B had negative HE4 (<120 pmol/L) but positive CA125(>100 U/mL), while 30 (15.7%) patients had negative CA125 but positive HE4.

## 4. Discussion

In the present study, ROMA and CPH-I performed similarly to identify EOC or MCO. The algorithms performed significantly better than tumor markers alone. ROMA index includes menopausal status, whose definition is not the same in all studies and may sometimes be unknown [15]. CPH-I is independent of menopausal status and includes age, which is easier to obtain [17]. The diagnostic performance of isolated tumor markers and probabilistic indexes was different in the subgroups of premenopausal patients, patients with inconclusive ultrasonographic diagnosis and for the detection of Stage I EOC.

### 4.1. Diagnostic Performance of Tumor Markers and Probabilistic Indexes in Overall Population

The authors of the *ESGO/ISUOG/IOTA/ESGE Consensus Statement on Preoperative Diagnosis of Ovarian Tumors* state that neither HE4 nor ROMA improve the discrimination between benign and malignant masses compared to HE4 alone [28]. Comparing the sensitivity and specificity of the tumor markers and the probability indexes with those reported in the literature is challenging [16,18,21], as the cutoff points used by different groups are not always the same, and neither are the comparison groups according to pathological findings (e.g., in some publications, borderline tumors or non-epithelial ovarian cancer are included in the malignancy group and compared to benign ovarian tumors [18,21]). The sensitivity and specificity values of ROMA and CPH-I shown in Table 3 are consistent with those previously reported. Moore et al. [29] reported sensitivity for ROMA of 94% at a set specificity of 75% at a cutoff point 12.9/27.8 (pre/postmenopausal), while in a meta-analysis by Dayyani et al. [16] a sensitivity of 87.3% and a specificity of 85.5% are reported (different cutoff values depending on the study). In the validation cohort of Karlsen et al., a sensitivity of 82.0% and specificity of 88.4% for CPH-I at a cutoff of 7 were found [17].

There are few studies in the literature comparing ROMA and CPH-I. The study of Minar et al. [18] reported a sensitivity and specificity of 71% and 88% for ROMA (cutoff point 13.1/27.7 for pre/postmenopausal) and of 69% and 85% for CPH-I (cutoff point 7). The study of Yoshida et al. [22] described sensitivity of 71.2% and specificity of 83.5% for ROMA (cutoff 13.1/27.7), and of 73.1% and 84.4% for CPH-I (cutoff point 7). In our study, sensitivity and specificity at these cutoff points are slightly higher. Similarly, Tu Tran et al. recently reported a sensitivity and specificity of 74.2% and 91.8% for ROMA (cutoff 16.5) and 87.1% and 78.5% for CPH-I (cutoff 1.89), respectively. The authors of the latter work did not find significant differences between the diagnostic performance of ROMA and CPH-I [23].

### 4.2. Diagnostic Performance of Tumor Markers in Specific Subgroups of Patients

In premenopausal women, HE4 performed significantly better than CA125 and was equivalent to ROMA or CPH-I, highlighting the value of this tumor marker in this subgroup of patients. This finding is supported by previous studies that suggest that in premenopausal women ROMA does not seem to have any advantage over the use of HE4 [30,31,32]. A recent meta-analysis retrieving data from 32 studies showed higher AUC and specificity of HE4 in premenopausal women, compared to CA125 and ROMA [33]. Other authors report higher AUCs for CPH-I, when compared to ROMA and HE4 in premenopausal women [23].

CA125 can raise in patients with endometriosis, thus differentiating ovarian endometriosis from EOC in premenopausal women has been a question of concern. HE4 is not usually above normal values in patients with endometriosis [11]. In our cohort, the majority of false positive results of CA125 were found in patients with endometriomas (96 patients), while only one patient with elevated HE4 harbored ovarian endometriosis. Patients with ovarian endometriosis accounted for 14% and 19% of false positive results of ROMA and CPH-I, which is to be expected considering that both probabilistic indexes include the value of CA125. A work on differential diagnosis of EOC and ovarian endometriosis in premenopausal woman suggests that HE4 has higher accuracy and AUC than CA125 and ROMA [34].

Most patients with false negative results of probabilistic indexes and tumor markers presented initial stages of ovarian cancer, whose preoperative diagnosis still remains challenging. In our cohort, probabilistic indexes performed similarly, and ROMA performed better than tumor markers alone for diagnosis of Stage I EOC. Moreover, in the analysis of the subgroup of patients with an inconclusive diagnosis by ultrasound, the ROMA model showed the highest AUC. This might suggest probabilistic models (especially ROMA) are particularly useful in cases whose diagnosis by ultrasound is challenging. Kaijser et al. [35] conclude that HE4 and ROMA, as secondary tests, do not seem useful for classification of adnexal tumors after subjective assessment with transvaginal ultrasonography by experienced Level III examiners. Further studies in these specific subpopulations are needed to confirm these data.

### 4.3. The Role of Isolated CA125 for Preoperative Assessment od Adnexal Tumors

HE4 performed better in terms of AUC, sensitivity and specificity than CA125. As mentioned before, in premenopausal women, HE4 was even equivalent to ROMA and CPH-I in ROC analysis. This raises the question of whether we can do without CA125 for preoperative assessment of patients with suspected ovarian cancer. Jacob et al. concluded that HE4 might be used alone, without the benefit of adding ROMA to the preoperative setting [36] and Montagnana et al. [32] concluded that the dual marker combination of HE4 and CA125 does not show better performance than HE4 alone in premenopausal women. However, our data show that nearly 10% of patients with EOC or MCO presented an isolated positivity of CA125 (with negative HE4). Moreover, except for premenopausal women (HE4 was equivalent to CPH-I and ROMA), and Stage I EOC (HE4 was equivalent to CPH-I), CPH-I and ROMA performed better than HE4 alone. Therefore, even if CA125 shows less specificity and sensitivity than HE4, it adds valuable information for the diagnosis of EOC or MCO.

### 4.4. Beyond CA125 and HE4: The Role of Novel Tumor Markers and Ultrasound

Some studies have evaluated the role of other biomarkers for diagnosis of ovarian cancer, with variable results. The OVA1 index is an FDA-approved multivariate index assay that has been considered a good triage tool for patients with ovarian masses requiring surgery, although its low specificity and its worse diagnostic performance in premenopausal women have raised some concerns [37,38,39,40,41]. A second generation multivariate index assay (Overa^®^) showed an improvement in terms of specificity (61.9%) with similar sensitivity (91.3%) to Ova1 [41]. Han et al. suggested in 2018 that a four biomarker panel including CA125, HE4, E-CAD, and IL-6 might be a potential tool for diagnosis of serous ovarian cancer at early stages [42]. Zhu et al. recently proposed a new algorithm including HE4, CA125 and thymidine kinase 1; the authors suggest that this algorithm has better diagnostic performance than ROMA, and report higher AUCs than those identified in our cohort, for both pre- and post-menopausal women [43]. In exchange, Moore et al. report that the addition of other biomarkers to HE4 and CA125 does not improve diagnostic performance [38]. Further validation studies are needed to confirm these results.

Ultrasound adds valuable information to preoperative assessment of adnexal masses, thus several models based on ultrasound features have been described, such as RMI, IOTA-LR1 and LR2 or ADNEX [44]. Notably, IOTA-LR1 and LR2 models have shown good diagnostic performance, with reported AUCs of 0.96 and 0.95 respectively, higher than those obtained for tumor-marker based models in our cohort [45]. ADNEX model combines ultrasound features with CA125, and has a sensitivity of 96.5%, a specificity of 71.3%, and a 0.94 AUC [46]. The *ESGO/ISUOG/IOTA/ESGE Consensus Statement on Preoperative Diagnosis of Ovarian Tumors* reports that ultrasound-based diagnostic models (IOTA simple rules or ADNEX) are preferable to CA125 level, HE4 level or ROMA [28]. In 2015, an algorithm including HE4, menopausal status and ultrasound findings was described, with higher sensitivity but less specificity than ROMA algorithm [47]. To the best of our knowledge, to date, no algorithms including ultrasonographic items and HE4 have been validated. Further studies are needed to determine whether a probabilistic model combining clinical information, ultrasound features and both HE4 and CA125 might offer the best diagnostic approach.

### 4.5. Strengths and Limitations of the Present Study

We believe that the most relevant limitation of the present study is the retrospective nature of the analysis. Patients were recruited in a single institution, which might be a limitation in terms of the external validity of the study. Nevertheless, we included a high number of patients from the same hospital, which allowed us to achieve a high degree of uniformity in the procedures performed (blood sample extraction and processing, pathologic analysis, ultrasound performance). To the best of our knowledge, this is the first single institution study that includes more than 1000 patients comparing the role of CPH-I and ROMA indexes for preoperative assessing of adnexal tumors. We expect that data arising from this study will help to improve diagnostic protocols for preoperative evaluation of adnexal masses. However, further prospective studies are needed to determine the best diagnostic algorithm.

## 5. Conclusions

Improving preoperative diagnosis of patients with ovarian malignancies is essential to enhancing their survival. CA125 and HE4, and the probabilistic indexes ROMA and CPH-I have been proposed as a tool for preoperative evaluation of patients with adnexal tumors. According to our data, ROMA and CPH-I perform significantly better than tumor markers alone to identify patients harboring ovarian malignancies in overall population. In premenopausal women, HE4 is equivalent to probabilistic indexes. ROMA performs better for the diagnosis of Stage I EOC and for patients harboring adnexal masses whose diagnosis by ultrasound remains inconclusive.

ROMA and CPH-I can be helpful to assess the risk of malignancy of adnexal masses, especially when expert ultrasound examination is not available or when the diagnosis is challenging (early-stage ovarian cancer, inconclusive ultrasonographic evaluation). An algorithm including CA125, HE4 and ultrasound features might improve the diagnostic approach. Further prospective studies are needed to determine the best diagnostic algorithm for patients with ovarian malignancies.

## Figures and Tables

**Table 1:** General characteristics of the study population.

	*N* = 1071		
Age (mean ± SD)	47.72 ± 16.22		
Menopausal status			
Premenopausal	629 (58.73%)		
Postmenopausal	442 (41.27%)		
Pathology			
Benign	778 (72.64%)		
Epithelial tumor	331 (42.5%)		
Endometrioma	210 (26.9%)		
Fibroids	56 (7.2%)		
Other sex cord-stromal tumors	4 (0.5%)		
Germ cell tumors	149 (19.2%)		
Fallopian tube lesions	24 (3.1%)		
Tubo-ovaric abscesses	4 (0.5%)		
Epithelial borderline	74 (6.91%)		
Epithelial ovarian cancer (EOC)	197 (18.39%)	**FIGO stage for EOC**	
Serous carcinoma	124 (62.94%)	FIGO Stage I	58 (29.44%)
Mucinous carcinoma	8 (4.06%)	FIGO Stage II	15 (7.61%)
Clear cell carcinoma	17 (8.63%)	FIGO Stage III	108 (54.82%)
Endometrioid Carcinoma	33 (16.75%)	FIGO Stage IV	16 (8.12%)
Undifferenciated Carcinoma	15 (7.61%)		
Metastatic cancer in the ovary	22 (2.05%)		

**Table 2:** ROC analysis of Ca125, He4, ROMA and CPH for diagnosis of EOC or MCO. Area under the curve (95% CI).

	All Patients	Inconclusive Diagnosis *	Premenopausal	Postmenopausal	Stage I EOC
CA125	0.873 (0.842–0.904)	0.810 (0.743–0.877)	0.759 (0.675–0.842)	0.933 (0.907–0.959)	0.810 (0.751–0.869)
HE4	0.909 (0.881–0.938)	0.844 (0.779–0.910)	0.863 (0.791–0.934)	0.905 (0.870–0.940)	0.856 (0.793–0.8869)
ROMA	0.939 (0.916–0.962)	0.893 (0.846–0.941)	0.866 (0.796–0.937)	0.956 (0.937–0.975)	0.909 (0.863–0.955)
CPH	0.936 (0.913–0.958)	0.876 (0.822–0.931)	0.860 (0.793–0.928)	0.955 (0.935–0.975)	0.901 (0.855–0.947)

* Inconclusive diagnosis of malignancy by ultrasound features using IOTA simple rules.

**Table 3:** Sensitivity, specificity, and predictive values of Ca125, He4, ROMA index and CPH-1 index at different cutoff points.

Parameter	Cutoff	Sensitivity (95% CI)	Specificity (95% CI)	PPV (95% CI)	NPV (95% CI)
CA125	100 U/mL	61.86 (55.21–68.09)	92.71 (90.73–94.29)	68.91 (62.07–75.02)	90.30 (88.11–92.11)
	35/65 U/mL *	74.42 (68.23–79.81)	80.70 (77.81–83.24)	50.21 (44.73–55.64)	92.30 (90.22–94.12)
	35/100 U/mL *	66.98 (60.44–72.92)	81.39 (78.58–83.90)	48.48 (42.86–54.15)	90.41 (88.07–92.32)
HE4	70 pmol/L	83.25 (77.31–87.88)	86.11 (83.40–88.43)	61.15 (55.11–66.87)	95.14 (93.22–96.53)
	120 pmol/L	69.11 (62.23–75.23)	96.29 (94.65–97.44)	83.02 (76.42–88.06)	92.23 (90.10–93.93)
	70/140 pmol/L *	70.70 (63.93–76.71)	93.5 (91.52–95.11)	74.2 (67.40–80.01)	92.40 (90.20–94.10)
ROMA	10%	94.24 (89.98–96.75)	72.71 (69.27–75.91)	47.91 (43.84–54.01)	97.85 (96.19–98.80)
	15%	91.10 (86.21–94.37)	84.62 (81.73–87.12)	62.14 (56.33–67.62)	97.17 (95.51–98.22)
	12.5/14.4% *	91.62 (86.83–94.78)	82.58 (79.57–85.23)	59.32 (53.63–64.77)	97.26 (95.60–98.31)
	13.1/27.7% *	84.80 (79.00–89.20)	89.0 (86.40–91.10)	68.10 (61.90–73.70)	95.50 (93.60–96.80)
CPH	1%	96.86 (93.32–98.55)	47.97 (44.26–51.70)	34.01 (30.15–38.09)	98.22 (96.17–99.18)
	3%	91.10 (86.22–94.42)	79.12 (75.91–82.01)	54.72 (49.23–60.14)	97.01 (95.21–98.12)
	5%	86.91 (81.39–90.97)	87.54 (84.86–89.79)	65.87 (59.82–71.45)	96.03 (94.20–97.29)
	7%	82.20 (76.16–86.97)	90.87 (88.49–92.80)	71.36 (65.06–76.93)	94.86 (92.90–96.309)

PPV: positive predictive value; NPV: negative predictive value * premenopausal/postmenopausal women.

## Data Availability

The data presented in this study are available on request from the corresponding author.

## Supplementary Materials

The following are available online at https://www.mdpi.com/article/10.3390/diagnostics12010226/s1, Table S1: ROC analysis of Ca125, He4, ROMA and CPH for diagnosis of EOC or MCO. *p*-values of the comparison between ROC curves. Table S2: false negative results for tumor markers and probabilistic indexes.
