# Supplementary material for: Comparison of HE4, CA125, ROMA and CPH-I for Preoperative Assessment of Adnexal Tumors
Source: Diagnostics (Basel). 2022 Jan 17;12(1):226. doi: 10.3390/diagnostics12010226 (PMC8774736; doi:10.3390/diagnostics12010226)
Supplement: Supplementary file 1 [file diagnostics-12-00226-s001.zip › diagnostics-1524606-supplementary.pdf]

Supplementary Material

Table S1. ROC analysis of CA125, HE4, ROMA and CPH for diagnosis of EOC or MCO. *p*-values of the comparison between ROC curves.

		Total		
	CA125	***HE4***	***ROMA***	***CPH***
AUC (95% CI)	*0.873 (0.842–0.904)*	*0.909 (0.881–0.938)*	*0.939 (0.916–0.962)*	*0.936 (0.913–0.958)*
CA125	-	*0.0294*	*0.0000*	*0.0000*
He4	*0.0294*	-	*0.0004*	*0.0053*
ROMA	*0.0000*	*0.0004*	-	*0.5994*
CPH	*0.0000*	*0.0053*	*0.5994*	-
		Premenopausal		
	CA125	***HE4***	***ROMA***	***CPH***
AUC (95% CI)	*0.756 (0.675–0.842)*	*0.863 (0.791–0.934)*	*0.866 (0.796–0.937)*	*0.860 (0.793–0.928)*
CA125	-	*0.0125*	*0.0000*	*0.0000*
He4	*0.0125*	-	*0.0005*	*0.0057*
ROMA	*0.0000*	*0.0005*	-	*0.6118*
CPH	*0.0000*	*0.0057*	*0.6118*	-
		Postmenopausal		
	CA125	***HE4***	***ROMA***	***CPH***
AUC (95% CI)	*0.933 (0.907–0.959)*	*0.905 (0.870–0.940)*	*0.956 (0.937–0.975)*	*0.955 (0.935–0.975)*
CA125	-	*0.1569*	*0.0075*	*0.0310*
HE4	*0.1569*	-	*0.0001*	*0.0001*
ROMA	*0.0075*	*0.0001*	-	*0.3882*
CPH	*0.0310*	*0.0001*	*0.3882*	-
		Inconclusive ultrasound according to IOTA simple rules		
	CA125	***HE4***	***ROMA***	***CPH***
AUC (95% CI)	*0.810 (0.743–0.877)*	*0.844 (0.779–0.910)*	*0.893 (0.846–0.941)*	*0.876 (0.822–0.931)*
CA125	-	*0.3863*	*0.0005*	*0.0037*
HE4	*0.3863*	-	*0.0316*	*0.1399*
ROMA	*0.0005*	*0.0316*	-	*0.0344*
CPH	*0.0037*	*0.1399*	*0.0344*	-
		Stage I EOC		
	CA125	***HE4***	***ROMA***	***CPH***
AUC (95% CI)	*0.810 (0.751–0.869))*	*0.856 (0.793–0.8869)*	*0.909 (0.863–0.955)*	*0.901 (0.855–0.947)*
CA125	-	*0.2410*	*0.0014*	*0.0001*
HE4	*0.2410*	-	*0.0099*	*0.0572*
ROMA	*0.0014*	*0.0099*	-	*0.6415*
CPH	*0.0001*	*0.0572*	*0.6415*	-

Table S2. False negative results for tumor markers and probabilistic indexes.

	CA125	*HE4*	*ROMA*	*CPH*
Total	*53 (100%)*	*38(100%)*	*8 (100%)*	*20 (100%)*
Serous carcinoma	*27 (50.94%)*	*16 (42.11%)*	*3 (37.50%)*	*6 (30.00%)*
Endometrioid carcinoma	*14 (26.42%)*	*8 (21.05%)*	*2 (25.00%)*	*7 (35.00%)*
Mucinous carcinoma	*2 (3.77%)*	*5 (13.06%)*	*3 (37.50%)*	*3 (15.00%)*
Clear cell carcinoma	*10 (18.87%)*	*9 (23.68%)*	*0 (0.00%)*	*4 (20.00%)*
Undifferenciated	*6 (11.32%)*	*6 (15.79%)*	*2 (25.00%)*	*4 (20.00%)*
Metastatic cancer in the ovary	*12 (22.64%)*	*15 (39.47%)*	*7 (87.50%)*	*10 (50.00%)*
